# Verteporfin exhibits YAP-independent anti-proliferative and cytotoxic effects in endometrial cancer cells

**DOI:** 10.18632/oncotarget.15614

**Published:** 2017-02-22

**Authors:** Venkata Ramesh Dasari, Virginia Mazack, Wen Feng, John Nash, David J. Carey, Radhika Gogoi

**Affiliations:** ^1^ Weis Center for Research, Geisinger Medical Center, Danville, PA, USA; ^2^ Henry Hood Center for Health Research, Geisinger Medical Center, Danville, PA, USA

**Keywords:** endometrial carcinoma, hippo pathway, verteporfin, YAP, organoids

## Abstract

Endometrial Carcinoma (EMCA) is the most common gynecologic malignancy and the fourth most common malignancy in women in the United States. Yes-associated protein (YAP) is a potent transcription coactivator acting via binding to the TEAD transcription factor, and plays a critical role in organ size regulation. Verteporfin (VP), a benzoporphyrin derivative, was identified as an inhibitor of YAP-TEAD interaction. We investigated the therapeutic efficacy and mechanism of VP in EMCA. The efficacy of VP on cell viability, cytotoxicity and invasion was assayed in EMCA cell lines. An organoid model system was also developed to test the effect of VP on apoptotic markers in an *in vitro* model system. Treatment with VP resulted in a decrease in cell viability, invasion and an increase in cytotoxicity of EMCA cells. These effects occurred as early as 15 minutes following treatment. Similarly, VP treatment versus vehicle control increased apoptosis in human organoid model systems. Quantitative RT-PCR, cDNA based RTPCR array analysis and western blotting were performed to investigate the mechanism of VP action. The cytotoxic and anti-proliferative effects appeared to be independent of its effect on YAP. Our results suggest that VP is a promising chemotherapeutic agent for the treatment of endometrial cancer.

## INTRODUCTION

Endometrial Carcinoma (EMCA) is the most common gynecologic malignancy and the fourth most common malignancy in women with an estimated 60,050 new cases and 10,470 deaths estimated in 2016 in the United States alone [1]. Of the two types of EMCA, Type 1 cancer accounts for approximately 80% and is characterized as estrogen dependent, estrogen receptor (ER) and progesterone receptor (PR) positive with endometrioid morphology and generally a favorable prognosis [2, 3]. Conversely, type 2 cancer is estrogen-independent, ER/PR negative, poorly differentiated with non-endometrioid (serous, clear-cell carcinoma) morphology and associated with a much poorer prognosis [2, 3]. There is evidence to suggest that the morphological and clinical differences between type I (endometrioid) and type II (non-endometrioid) endometrial cancers are mirrored in their genetic alterations [4], given that they harbor mutations affecting distinct genes and signaling pathways [5].

Ongoing trials are aimed at identifying those patients at highest risk of recurrence and their response to therapy (including chemotherapy, chemoradiation therapy, and molecular targeted therapies) to optimize survival and quality of life [6]. Molecularly targeted agents including mTOR inhibitors and anti-angiogenic agents have been used in limited clinical trials in EMCA [5], although early results of clinical trials revealed limited efficacies of these agents. It should be noted, that these clinical trials did not stratify patients according to histological subtype, presence of the therapeutic target or other biomarkers of response, and patients enrolled in clinical trials were heavily pretreated [7]. We suggest that successful treatment of EMCA will require individualization of therapies based on the molecular and/or genetic make-up of the EMCA cells.

Yes-associated protein (YAP) is a transcriptional co-activator and the main downstream target of the HIPPO pathway [8]. YAP promotes cell proliferation, inhibits cellular apoptosis, and also promotes an epithelial-mesenchymal transition (EMT) in a number of tumor types [9–12]. YAP is amplified in a number of human cancers including breast, esophageal [13], hepatocellular [14, 15], malignant mesothelioma [16, 17], medulloblastoma [18] and ovarian cancers [9, 11]. Several reports show that gene amplification and epigenetic modulation of the YAP locus and that of its binding partner Tafazzin (TAZ) loci in cancer are significant for the development and sustainability of neoplasia [10, 19, 20]. Inhibition of the HIPPO pathway leads to YAP activation, nuclear localization and expression of target genes that promote cell proliferation. Conversely, HIPPO pathway activation leads to phosphorylation on specific serine residues to confine YAP/TAZ in the cytoplasm for subsequent degradation [21, 22]. Nuclear YAP/TAZ interacts with the TEAD family of transcription factors to induce expression of genes that promote cell proliferation and inhibit apoptosis. We have previously shown that YAP nuclear expression is a marker of poor prognosis and a potential therapeutic target in EMCA [23].

Verteporfin (VP) [24], an FDA approved drug used in photodynamic therapy (PDT) for macular degeneration was recently identified as an inhibitor of YAP-TEAD binding [25]. VP binds to YAP and changes its conformation, thereby abrogating its interaction with TEAD2 [25]. VP also inhibits YAP induced liver overgrowth in a transgenic mouse model, suggesting a pharmacological strategy for regulating the transcriptional activities of YAP [25]. We tested the efficacy of VP as a chemotherapeutic agent for the treatment of EMCA.

## RESULTS

### VP inhibits cell viability and invasion and increases cytotoxicity of EMCA cells

**Figure 1:**
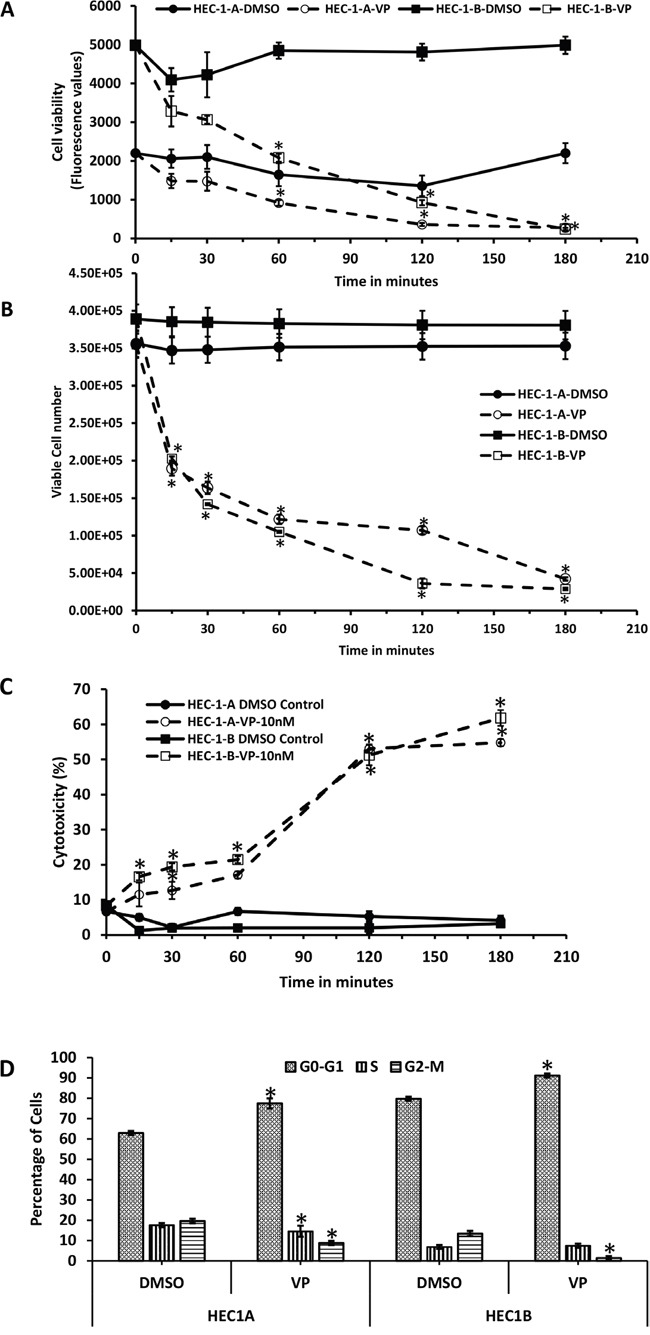
Anti-proliferative effects of VP on EMCA cells **A**. Cell Viability assay after treatment of HEC-1-A and HEC-1-B cells (10,000 each well) with DMSO or VP at 10 nM concentrations from 0-3h. Error bars indicate Mean ±SEM. n=4. *Statistically significant at p<0.05. (p = 0.047, 0.0089, and 0.0001 for HEC-1-A and 0.0001, 0.0001 and 0.0001 for HEC-1-B respectively). Experiment is repeated 4 times with at least 3 replicates. **B**. Trypan blue assay measuring viable cell number at specific time periods. EMCA cells (10,000 each well) were treated with DMSO or VP at 10 nM concentrations after 0-h. Cells that excluded trypan blue were measured. Error bars indicate Mean ±SEM. n=4. *Statistically significant at p<0.05. (p=0.0019, 0.0001, 0.0001, 0.0001 and 0.0001 for HEC-1-A and 0.0017, 0.0001, 0.0001, 0.0001 and 0.0001 for HEC-1-B respectively). Experiment is repeated 3 times with at least 3 replicates. **C**. LDH cytotoxicity assay of HEC-1-A and HEC-1-B cells after treatment with VP at 10 nM for 0-3h. *Statistically significant at p<0.05. (p=0.0073, 0.00001, 0.0001 for HEC-1-A and 0.0008, 0.0004, 0.0001, 0.0002, 0.00004 for HEC-1-B). Error bars indicate Mean ±SD. n=8. Experiment is repeated 3 times with at least 3 replicates. **D**. Flow cytometric analysis of EMCA cells after VP treatment for 3h at 10 nM. n=3. Error bars indicate Mean ±SEM. *statistically significant at p<0.05. (p=0.0233, 0.0139 for HEC-1-A and 0.012 and 0.0001 for HEC-1-B respectively). Experiment is done with at least 3 replicates for each cell line.

To determine whether VP affected the behavior of EMCA cells in culture, we performed pilot experiments at varying doses (2, 5 and 10 nM) and time points (15 minutes to 24h). A dose of 10 nM was chosen as the concentration for all the experiments shown based on the cell viability and cytotoxicity assays. We used two type 1 EMCA cell lines HEC-1-A and HEC-1-B to test the effect of VP in EMCA. Cell viability (as measured by fluorescence) was performed following VP treatment. By 3 hours, 10 nM VP induced a statistically significant decrease in cell viability as measured by decreased fluorescence of remaining viable cells compared to the DMSO control in both cell lines (Figure 1A) with no significant rebound by 6 hrs. (data not shown). To evaluate how quickly this effect occurs, cell viability was measured at 15 min, 30 min, 1h, 2h and 3hrs. A decrease in cell viability can be seen within 15 min following VP treatment.

We next assessed the cytotoxic effects of VP by measuring LDH release and trypan blue assay exclusion as a measure of cell viability. To quantify viable cell number, a trypan blue assay was performed at 15 min, 30 min, 1, 2 and 3hrs and trypan blue excluded cells measured. Our data show a statistically significant decrease in viable cell numbers within 15 min of VP treatment in both EMCA cell lines (Figure 1B). VP induced a statistically significant cytotoxic effect as measured by the LDH assay in both EMCA cell lines (Figure 1C). To determine whether the decrease in cell viability induced by VP was caused by an inhibition of the cell cycle, flow cytometric analysis was performed and showed VP induced cell cycle arrest at G_0_-G_1_ at 3hrs. in both cell lines (Figure 1D) with 77.44% of HEC-1-A cells compared to 62.87% in control cells and 91.14% of HEC-1-B cells in G_0_-G_1_ phase compared to 79.7% in control cells.

**Figure 2:**
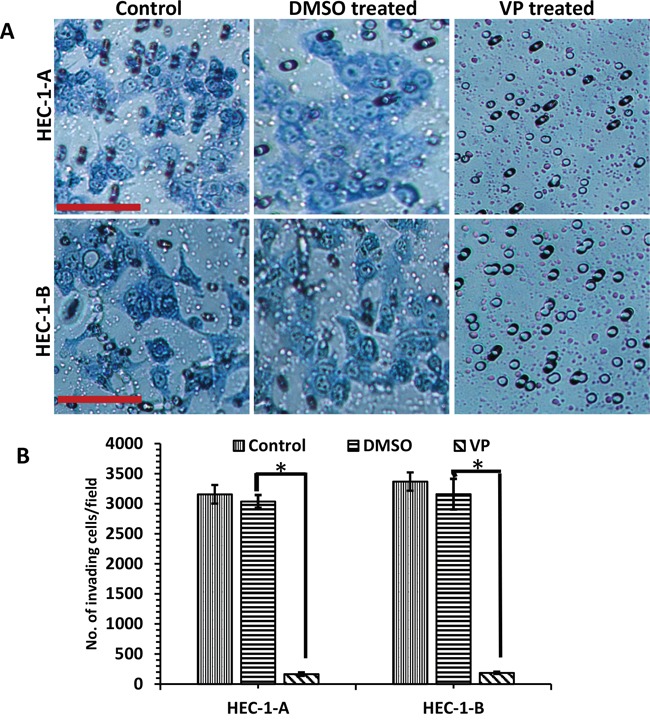
Inhibition of invasion of EMCA cells by VP **A**. EMCA cell lines (each 100,000) were treated with DMSO, VP or untreated at 10 nM for 3h and were allowed to invade through the Matrigel for 36h. Transwell cell inserts with 8μm pores were used. n=9. Bar = 20x. **B**. Quantitative estimation of matrigel invasion assay. Error bars indicate Mean ±SEM. *Statistically significant at p<0.05. (p=0.0001 for HEC-1-A and 0.0001 for HEC-1-B). Experiment is repeated 3 times with at least 3 replicates for each cell line.

We next investigated the effect of VP on EMCA cell invasion. To ensure that equal numbers of viable cells were plated in the VP and DMSO group, cells were either untreated or treated for 3 hrs. with either DMSO or VP, counted and an equal number of viable cells were plated on Boyden chambers coated with matrigel. Our results demonstrate a significant decrease in invasion in the VP treated group compared to control DMSO treated and untreated cells (Figure 2A). The inhibitory effect of VP was significant in both EMCA cell lines with 91.56% inhibition in HEC-1-A (two tailed t-test; p≤0.0001) and 94.10% inhibition in HEC-1-B cells (two tailed t-test; p≤0.0001) (Figure 2B). These results demonstrate that VP induces cytotoxicity, decreases cell viability, and decreases invasion of EMCA cells.

**Figure 3:**
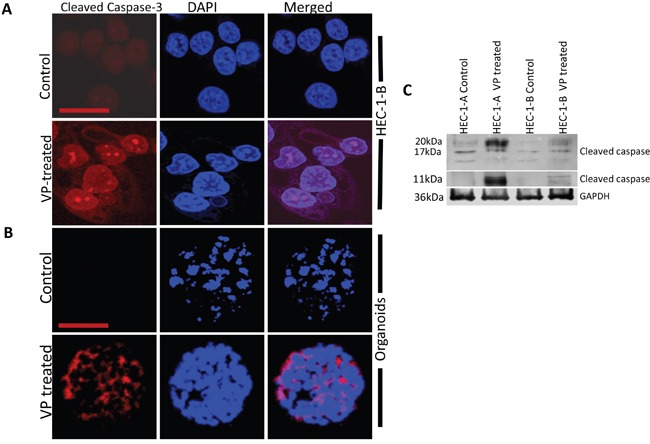
VP induces caspase-3 mediated apoptosis in HEC-1-B Cells and organoids Confocal images of **A**. HEC-1-B cells and **B**. organoid model system (#1077), which were subjected to immunofluorescence detection for cleaved caspase-3 after VP treatment at 10 nM for 3h. Cleaved-caspase-3 (anti-rabbit) is conjugated with goat anti-rabbit Alexa flour secondary antibody. Bar for HEC-1-B = 63x and Bar for organoids is =20x. **C**. Equal amounts of proteins (40μg) from untreated and treated EMCA cells were loaded on 14% gels and transferred onto nitrocellulose membranes, which were then probed with respective antibodies. GAPDH was used a positive loading control. n=3.

To further examine the apoptotic effects of VP, we analyzed the expression of cleaved caspase-3 by immunofluorescence in HEC-1-B cells and in an *in-vitro* organoid model of cells isolated from patient specimens of grade 1 EMCA. The *in vitro* organoid model is a more physiological 3D model that facilitates investigation of a range of *in vivo* biological processes including tissue renewal, stem cell/niche functions and tissue responses to drugs, mutation or damage [26]. The organoids were CK7+ve and CK20-ve consistent with endometrioid adenocarcinoma (Supplementary Figure 6). Cleaved caspase-3 was highly expressed in the EMCA cells and organoids after 3 hours of VP treatment (Figures 3A, 3B). We observed similar expression of cleaved caspase-3 in the VP treated HEC-1-A cells and in organoids isolated in a second patient specimen (Supplementary Figure 1), indicating that VP produces similar effects in a heterogeneous tumor model more closely representing the human environment. Western blot analysis confirms cleaved caspase-3 expression following VP treatment in EMCA cells (Figure 3C). VP also induces phenotypic changes in EMCA cell lines after 3h and 6h treatments. Loss of actin filaments and condensation of nuclear materials were prominently observed after VP treatments (Supplementary Figure 2).

### VP and HIPPO pathway

**Figure 4:**
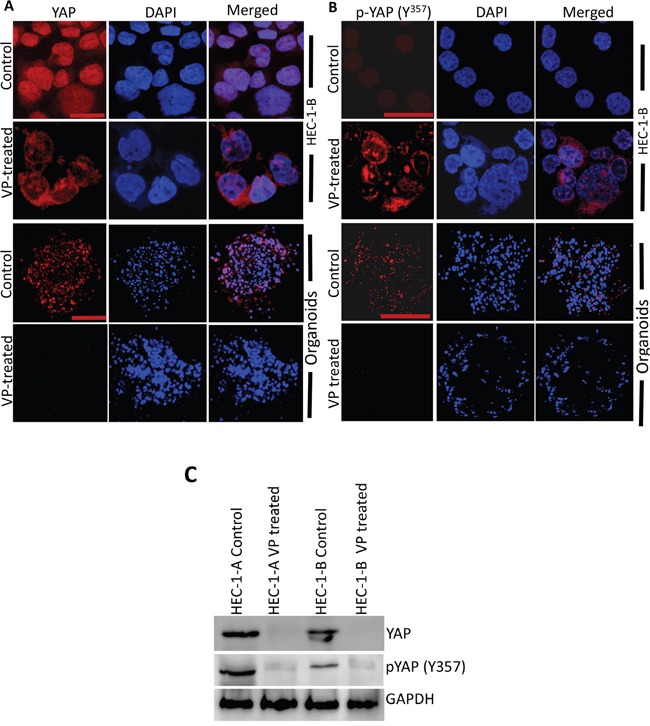
VP downregulates YAP and phospho-YAP of HEC-1-B Cells and organoids Confocal images of HEC-1-B cells and organoids which were subjected to immunofluorescence detection of **A**. YAP and **B**. phospho-YAP after VP treatment. YAP and phospho-YAP are conjugated with goat anti-mouse and goat anti-rabbit Alexa flour secondary antibodies respectively. (A) Upper panel bar=63x and lower panel bar = 20x. (B) Upper panel bar=63x and lower panel bar = 20x. **C**. Equal amounts of proteins (40μg) from untreated and treated EMCA cells were loaded on 10% gels and transferred onto nitrocellulose membranes, which were then probed with YAP and phospho-YAP (y357) antibodies. GAPDH was used a positive loading control. n=3.

Since VP was first identified through a YAP inhibition screen, we next sought to understand the effects of VP on YAP and the HIPPO pathway in EMCA. To study the effect of VP on YAP expression, we performed immunofluorescence analysis of HEC-1-A and HEC-1-B cells after 10 nM VP treatment for 3h. In control cells, YAP is mostly nuclear and very little phospho-YAP (Y^357^) is observed. (Figure 4A, 4B, Supplementary Figure 3). VP treatment decreased total YAP and phospho-YAP staining and reduced the amount of nuclear YAP in EMCA cells. Similar results were observed after VP treatment of organoids (Figures 4A, 4B). These results were further confirmed by Western analysis of cell lysates of EMCA cells after treatment with VP (Figure 4C), suggesting that VP inhibits YAP expression and YAP signaling in EMCA cells.

**Figure 5:**
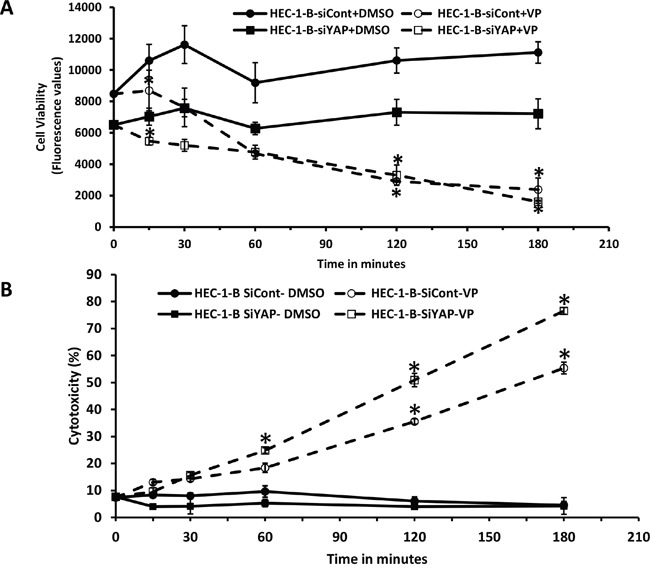
Mechanism of action of VP is independent of YAP in EMCA cells **A**. Cell viability assay after treatment of siYAP-HEC-1-B cells (10,000 each well) with VP at 10 nM concentrations after 0h to 3h treatment. Experiment is repeated 3 times with at least 3 replicates for each cell line. Error bars indicate Mean ±SEM. *statistically significant at p<0.05. (p =0.0308, 0.0107 and 0.0051 for respective time points). **B**. LDH cytotoxicity assay after treatment of siYAP-HEC-1-B cells (10,000 each well) with VP at 10 nM concentrations after 0h to 3h treatment. For all these tests n=3. *Statistically significant at p<0.05. Error bars indicate Mean ±SEM. (p= 0.0493, 0.0317, <0.001 and 0.00002 for siCont-VP and 0.001, <0.0001, <0.0001 for siYAP-VP respectively). Experiment is repeated 3 times with at least 3 replicates for each cell line.

We next asked whether the effect of antiproliferative and cytotoxic effects of VP on EMCA cells occurred through the HIPPO pathway. To study this, HEC-1-B cells were transiently transfected with a YAP-specific siRNA (siYAP) or control siRNA (siCont) and viability and cytotoxicity assays performed. Similar to the previous results, a statistically significant decrease in cell viability was noted with VP treatment in siYAP cells compared to siCont cells. However, this appeared to be independent on YAP expression (Figure 5A). Maximum cytotoxic effect was seen with VP treatment in siYAP cells compared to VP treatment of siCont cells, suggesting an additive effect of YAP downregulation in EMCA cells (Figure 5B). Similar results were seen in HEC-1-A EMCA cells (data not shown). Thus, although, VP reduced YAP levels and inhibited YAP activity, our data suggest the YAP-independent anti-proliferative and cytotoxic effects of VP.

### Mechanism of VP action on the HIPPO pathway

**Figure 6:**
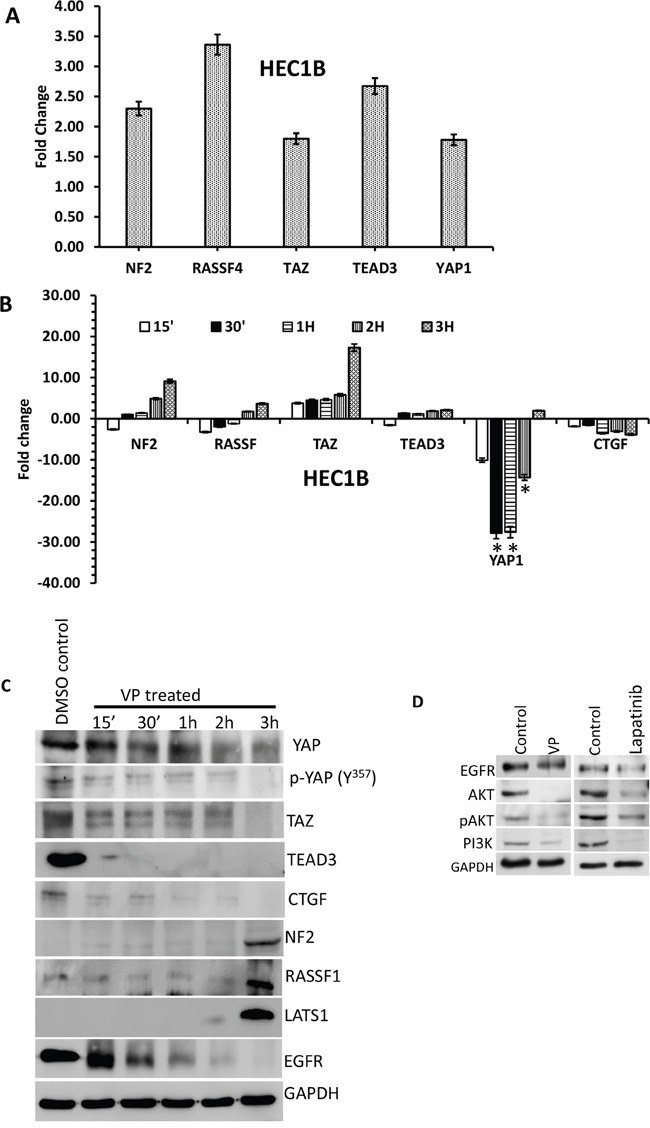
Effect of VP on HIPPO pathway genes of EMCA cells **A**. Effect of VP on HIPPO pathway of EMCA cells: cDNA RTPCR arrays were run after treatment of endometrial cancer cells with VP at 10 nM concentration for 3h. Control cells were treated with DMSO (vehicle). n=2 for each cell line and treatment. *Statistically significant at p<0.05. Error bars indicate Mean ±SEM. **B**. RTPCR analysis of selected HIPPO pathway genes. All the genes were normalized to the expression of GAPDH, β-actin, PGK1, LDHA and PPIH. *Statistically significant at p<0.05, by one-way ANOVA. DMSO control vs VP treated samples. Error bars indicate Mean ±SEM. p values are 0.0459, 0.0460 respectively. For each gene, duplicates were performed from 3 different samples for each treatment. n>6. **C**. Western blot time course of VP effect on YAP-mediated signaling molecules. Equal amounts of proteins (40μg) from untreated and treated (10 nM VP, 0 to 3 hrs.) EMCA cell lysates were loaded on 8% to 10% gels and transferred onto nitrocellulose membranes, which were then probed with respective antibodies. The westerns were run on separate blots. They were reprobed with GAPDH which was used a positive loading control. n=3. **D**. Western blots showing VP and Lapatinib effects on EGFR-mediated signaling molecules. Equal amounts of proteins (40μg) from untreated and treated (10 nM VP, 15 minutes; Lapatinib 20μM, 1h.) HEC-1-B cell lysates were loaded on 8% to 10% separate gels and transferred onto nitrocellulose membranes, which were then probed with respective antibodies. They were reprobed with GAPDH which was used a positive loading control. n=3.

To elucidate the effects of VP on the HIPPO/YAP pathway in EMCA cells, a cDNA RTPCR array for 84 HIPPO pathway genes was run following 3 hrs. of DMSO/VP treatment from HEC-1-A and HEC-1-B cells (Supplementary Table 1). VP upregulated 27 genes and downregulated 12 genes in HEC-1-A while in HEC-1-B, 21 genes were upregulated and 3 genes were downregulated by VP treatment. Supplementary Table 1 shows the most prominent gene expression changes induced by VP treatment. Tumor promoter oncogenes like CCNE2 and FAT1 were downregulated; whereas most of the tumor suppressor genes were upregulated by VP treatment. Figure 6A represents the fold changes of selective genes by PCR array after 3 hrs. of VP treatment in HEC-1-B cells. These genes were subsequently confirmed in a time course by quantitative PCR (Figure 6B). The expression levels of EGFR have been decreased and LATS1 are increased in VP treatments (Supplementary Figure 4). We additionally evaluated expression of CTGF, a direct YAP transcriptional target gene. Our data confirm that VP inhibits both RNA and protein expression of CTGF.

Western analysis of HIPPO pathway signaling molecules beginning at 15-minute time-point were performed. We observed a progressive decline in YAP expression along with its binding partner TEAD and TAZ with VP treatment. Increased expression of the HIPPO upstream proteins, LATS1 and NF2 correlated with decreased YAP expression (Figure 6C). These data suggest that the effect of VP occurs on the upstream mediators of the HIPPO pathway. We next sought to evaluate alternate mechanisms of VP action in EMCA. Based on the previously published data by Song et al., [27] that demonstrated YAP induced EGFR expression in esophageal cancer, we sought to explore the possible mechanism of VP action through EGFR in EMCA (Figure 6C). We observed that both YAP and EGFR are highly expressed in untreated EMCA cells. Both YAP and EGFR are downregulated by VP in a time-dependent manner with a more rapid downregulation of EGFR by 30 minutes. VP further inhibited downstream EGFR pathway proteins Akt and PI3K. To compare the effect of VP, we treated HEC-1-B cells with Lapatinib, a dual inhibitor of both EGFR and HER2 tyrosine kinase activity [28]. We observed that the inhibition of EGFR pathway by VP is comparable to the mechanism of action of Lapatinib (Figure 6D). It has been previously reported that VP exhibited *in vivo* selectivity for killing tumor cells in part by impairing the global clearance of high-molecular weight oligomerzed proteins, particularly p62 (a sequestrome involved in autophagy) and Stat3 [29, 30]. However, in our studies, we observed that VP is able to induce sequestration in p62 alone, but not in Stat3 (Supplementary Figure 5).

## DISCUSSION

Our data demonstrate the potential therapeutic efficacy of VP in EMCA. VP is a second generation potent photosensitizer clinically used in the photodynamic therapy of ocular neovascularization. VP is lipophilic and is more readily taken up by malignant and neo-vascular endothelial cells. When delivered i.v., the liposomal preparation of VP (Visudyne™) binds to the LDL receptor and undergoes rapid endocytosis. It's characteristics of selective uptake in rapidly proliferating cells, IV administration and limited toxicity at least in AMD in an animal model makes VP a promising drug to study for the treatment of EMCA. Although it was first identified as a photosensitizer, Liu-Chittenden et al identified VP in a drug screen for YAP/TEAD inhibitors [25].

Our interest in VP arose from a previous publication from our lab identifying nuclear YAP expression as a prognostic and a potential therapeutic target in EMCA [23]. We have shown previously that YAP inhibition decreases cell growth by 24hrs which we hypothesize is due to the time needed for down regulation of YAP by siRNA. The rapid cytotoxic effect of YAP in EMCA cells described here appears to be independent of its inhibition of YAP. YAP independent effects of VP have been previously described [29, 31]. Zhang et al described the efficacy of VP in a colorectal model that was independent of YAP inhibition [31]. Based on our data, we suggest that a combinatorial approach- one which combines YAP inhibition and VP may be the optimal approach to the treatment of patients with EMCA. Interestingly, our data demonstrate that a small percentage of EMCA cells remain resistant to VP treatment at the 3 hr. time point. We are in the process of developing VP resistant cell line to study the effect of combination therapy for maximum cytotoxicity.

To test the effects of VP on a model system more closely resembling *in vitro* conditions; we developed a unique organoid 3D model system with tumor cells isolated from a Type 1 EMCA patient tissue. VP-treated organoids had less expression of YAP and phospho-YAP and higher expression of cleaved caspase-3, suggesting that VP induces apoptosis and more inactive YAP in the cells of organoids. We also observed that many fewer viable organoids remain following VP treatment (data not shown). We found that VP exhibits anti-proliferative and apoptotic inducing activities of EMCA cells *in vitro*. The organoid model system may be an important method to study the various combinatorial approaches and doses prior to animal or human subject treatment.

We hypothesize that one possible mechanism for VP effect may occur through its downregulation of EGFR. EGFR plays key roles in essential cellular functions including proliferation and migration. High EGFR expression has been correlated with higher grade disease and lower disease free survival in EMCA [32]. The Gynecologic Oncology Group (GOG) has recently published phase 2 data on Lapatinib, a tyrosine kinase inhibitor of EGFR and HER2 in persistent or recurrent EMCA [33]. Our results have shown that both YAP and EGFR are downregulated in EMCA cell lines after treatment with VP, which ultimately inhibits growth and proliferation. Consistent with our results, Song et al., [27] recently demonstrated that VP effectively inhibits both YAP and EGFR protein levels and its downstream signaling and synergistically inhibit tumor cell growth *in vitro* and *in vivo* in esophageal cancer. Konecny et al., [34] reported concentration-dependent anti-proliferative effects of Lapatinib in several endometrial cancer cell lines tested, but effects varied significantly between individual cell lines. It was shown that Lapatinib suppresses MMP1 through EGFR and HER2, and their downstream ERK and AKT signaling pathways [35]. We are currently investigating the interaction of the HIPPO pathway and EGFR signaling. Based on our data, we suggest that combination therapy of VP and an EGFR inhibitor may show synergistic effects on EMCA cell lines.

In conclusion, our study shows that VP had the following effects on EMCA cell lines (1) VP inhibited viability of EMCA cells *in vitro*; (2) VP blocked the cell cycle progression of EMCA cells at the G_0_-G_1_ stage; and (3) VP induced apoptosis in EMCA cells and organoids. For the first time, we are able to show the effects of VP on endometrial cancer cell lines and the 3D model of organoids. Based on our preliminary *in vitro* results, we propose the use of VP as a potential therapeutic drug for the treatment of EMCA either as a single agent or in combination therapy. A detailed analysis of the mechanism and efficacy of VP in EMCA cell lines and in animal models is ongoing.

## MATERIALS AND METHODS

### Patients and tissue samples

Following approval by the institutional review board at Geisinger Medical Center (GMC) (IRB Protocol #2011–0163) EMCA tissues were obtained from patients undergoing hysterectomy. Macroscopic and microscopic classifications of tumors were based on the International Federation of Gynecologist and Obstetricians (FIGO) staging system [36].

### Organoids obtained from Patient specimens

Tumor tissue was obtained at time of hysterectomy. Cells were isolated from patient tissue samples after papain digestion using PDS kit from Worthington (Cat. No. LK003150). Tissue was minced into small pieces and placed in the Papain solution with DNase and incubated at 37°C with constant agitation for 30 min. Then the mixture was triturated with 10 ml pipette and 1 ml pipette tips. The cloudy cell suspension was passed through 40 μm cell strainer and centrifuged at 1000 rpm for 5 min at 4°C. The cell pellet was resuspended in 3 ml of EBSS containing albumin-ovomucoid inhibitor and DNase. The cell suspension was carefully layered on the top of 5 ml of albumin-inhibitor solution forming a discontinuous density gradient and centrifuged at 70xg for 6 min at 4°C. Dissociated cells pellet at the bottom of the tube, and membrane fragments remain at the interface. The pelleted cells were grown in 4-well chamber slides coated with Cultrex^®^ BME 2 RGF (ORGANOID MATRIX) PathClear^®^* (Amsbio, Cat. No. 3533-010-02). Cells were grown in primary cell medium composed of DMEM 1x supplemented with EGF (20 ng/mL), basic FGF (10 ng/mL), insulin (50μg/mL), BSA (0.4%) and 1% antibiotic/antimycotic solution. We plated 5000 cells in each well and organoids were differentiated after 3 days. Medium was replaced every 3 days and the organoids were passaged using an organoid harvesting kit from Amsbio (Cat. No. 3448-020-K). Using this technique, we were able to obtain organoids from 60% of patient samples. We used two different organoids obtained from two different patient samples in this study (#1002 and #1007). Organoids were characterized by cytokeratin 7 (CK7) and cytokeratin 20 (CK20) markers.

### EMCA cell lines and culture conditions

HEC-1-A (ATCC HTB112) and HEC-1-B (ATCC HTB113) were obtained from the American Type Culture Collection (ATCC) (Manassas, VA). HEC-1-A cells were cultured in McCoy's 5A medium (ATCC, Manassas, VA) supplemented with 10% (v/v) fetal bovine serum (FBS) (Thermo Fisher Scientific, Waltham, MA), HEC-1-B in Eagle's minimum essential medium (EMEM) (ATCC, Manassas, VA) supplemented with 10%(v/v) FBS. Antibiotics (10 units/ml of penicillin and 10 mg/ml of streptomycin) were added to all culture media. Both cell lines were incubated at 37°C in a humidified atmosphere containing 5% carbon dioxide.

### Verteporfin (VP) treatment

Verteporfin (Sigma, Cat. No. SML0534) was dissolved in DMSO and added to the medium for a final concentration of 2 nM, 5 nM or 10 nM for varying periods of 15 minutes, 30 minutes, 1h, 2h and 3h. Equal concentration of DMSO was added to the control cells.

### Lapatinib treatment

Lapatinib (Sigma, Cat. No. CDS022971) was dissolved in DMSO and added to the medium for a final concentration of 20μM for 1h. Equal concentration of DMSO was added to the control cells.

### Trypan blue exclusion test of cell viability

EMCA cells were cultured in 60 mm plates (each with 100,000 cells). Cells were treated with DMSO or VP at 10 nM after 2 days. The cells were harvested at the given time periods (0 to 3h) and counted using Cellometer AutoT4 (Nexcelom Bioscience).

### Measurement of cell viability by CellTiter-Blue cell viability assay

The CellTiter-Blue^®^ Assay (Promega) is based on the ability of living cells to convert a redox dye (resazurin) into a fluorescent end product (resorufin). EMCA cells were plated in 96-well microtiter plates at a final concentration of 10000 cells/well. Following treatment with either DMSO or VP for different periods (0 to 3h), CellTiter-Blue reagent was added and the plates were incubated at 37 °C for 1h and fluorescence read at 560/590 nm.

### Measurement of cytotoxicity by LDH assay

Cytotoxicity was evaluated by measuring lactate dehydrogenase (LDH) activity released in the media after VP exposure at different time points (0 to 3h) using the Pierce LDH Cytotoxicity assay kit (Thermo Scientific). The experiment was performed as per manufacturer's instructions in 96-well plates (each well 10000 cells) and quantitated by measuring wavelength absorbance at A_490_nm and A_680_nm.

### Western blot analysis

Cells were treated with either DMSO or VP (10 nM) for various time periods. After the treatment period, cells were lysed in RIPA buffer (Boston Bioproducts, Cat. No. BP-115DG) supplemented with protease and phosphatase inhibitors and subjected to SDS-PAGE. Samples were separated electrophoretically on 8% to 14% gels, electroblotted onto nitrocellulose membrane (Bio-Rad), blots were blocked at room temperature for 1 h in 5% (w/v) milk in phosphate-buffered saline and incubated overnight at 4°C with primary antibodies. Details of primary and secondary antibodies used in the study are explained in Supplementary Tables 2 and 3. Protein bands were visualized with an enhanced chemiluminescence substrate (Pierce Biotechnology) and detected using LAS-3000 (Fujifilm, Tokyo, Japan).

### Immunofluorescence and imaging

Cultured cells and organoids (after treatment with either DMSO or VP at 10 nM for 3h) were washed with PBS and fixed in 4% buffered formalin. After permeabilization with 0.03% Triton X-100 in PBS, cells were blocked in goat serum in PBS and incubated with respective antibodies (1:100) at 4°C overnight, followed by Alexa Fluor as secondary antibodies for 30 min at room temperature. After being mounted with 4, 6-diamidino-2-phenylindole (DAPI) for nucleus staining, cells were examined using a Nikon fluorescence microscope or Zeiss confocal microscope.

### Short interfering RNA (siRNA) treatment

The siRNA targeting the YAP gene (sc-38637; Santa Cruz) was used for downregulating YAP. The control siRNA (sc-37007; Santa Cruz) was used as a negative control. Each siRNA (37.5 nM) was transfected into EMCA cells (70% confluency) using Lipofectamine3000 (Invitrogen) according to the manufacturer's instructions. After 48 to 60 hours of transfection, the cells were harvested and used for various experiments. The knockdown of the target gene was verified by western blotting.

### RNA extraction and quantitative real time PCR

All primer sequences were determined using established human GenBank sequences. Primer sequences were designed using PrimerQuest (IDT) software. For Real-time polymerase chain reaction (RT-PCR) analysis and RT-PCR based array analysis (RT2 Profiler PCR Array, Qiagen) total RNA was isolated from control and VP treated EMCA cells. Total cellular RNA was extracted with RNeasy kit (Qiagen). We used RNA whose A260: A280 ratio is greater than 2.0. Total RNA was reverse transcribed into first strand cDNA using iScript cDNA Synthesis Kit (Bio-Rad), as per the manufacturer's instructions. The details of the primer sequences were given in Supplementary Table 4. Quantitative analysis of genes was done by SYBR green based real-time PCR using Applied Biosystems Real-Time PCR Detection System. Each sample was measured in triplicate and normalized to the reference GAPDH/β-actin/PGK1/LDHA/PPIH gene expression. ΔCT and ΔΔCT values were calculated and the fold change in the test gene expression was finally calculated (37). A statistical evaluation of RT-PCR results was performed using one-way analysis of variance (ANOVA) to compare test gene expression between control and VP treated cells.

### RTPCR array analysis

We used RNA isolated from EMCA cells treated with VP at 10 nM for 3h. For the HIPPO signaling pathway finder RT2 Profiler PCR Array (Cat. No. APRN-014A, Qiagen) expressing a panel of 96 primer sets of 84 relevant, pathway-focused genes, plus five housekeeping genes and three RNA and PCR quality controls. Real time PCR was done with following conditions: one cycle of 95 °C for 10 min and 40 cycles of 95°C for 15s and 60°C for 1 min. Data were exported to Excel files and analyzed using SuperArray RT2 Profiler PCR Array Data Analysis Template v3.0. The cut-off induction determining expression was 2.0 or − 2.0 fold changes. Genes, which suited the above criteria, were considered to be upregulated or downregulated. These experiments were performed in duplicate or triplicate as mentioned in the figure legends.

### Invasion assay

Transwell invasion assays were carried out using 24-well BioCoat cell culture inserts (BD Biosciences). The upper surface of 6.4-mm diameter filters with 8μm pores were precoated with extracellular matrix coating (Matrigel). After treatment with either DMSO or VP (10 nM, 3h), cells were washed twice with sterile 1x PBS to remove the dead cells, harvested and counted using Cellometer AutoT4 (Nexcelom Bioscience) counter. 100,000 viable cells in serum-free medium were seeded on to the upper chamber of each insert, with complete medium added to the bottom chamber. Following 36h of incubation, invasive cells on the lower surface of the filters were fixed and stained with the Differential Quik Stain Kit (Electron Microscopy Sciences), and counted.

### Cell cycle analysis

Cells were trypsinized after treatment with either DMSO or VP (10 nM, 3h) followed by incubation in a staining buffer (0.1% of Triton X-100, 0.2 mg/ml RNase A, and 40 mg/ml Propidium iodide in PBS). Cells were analyzed for DNA content using Beckman Coulter, Cytomics FC 500 (Brea, CA).

### Statistical analysis

All experiments were repeated at least 3 times (with triplicates) unless otherwise noted. Data are presented as Mean ± SEM unless otherwise noted. Data were analyzed for significance using one-way analysis of variance (ANOVA) using Graph Pad Prism software or MS Excel 2013. Results were considered statistically significant at a p<0.05 (DMSO treated vs VP treated).

## SUPPLEMENTARY MATERIALS FIGURES AND TABLES



## Abbreviations

AKT: AKT Serine/Threonine Kinase
AMD: Age-Related Macular Degeneration
bFGF: Basic Fibroblast growth factor
BSA: Bovine Serum Albumin
CCNE1: Cyclin E1
CK20: Cytokeratin 20
CK7: Cytokeratin 7
DAPI: 4′,6-Diamidino-2-Phenylindole, Dihydrochloride
DMEM: Dulbecco's Modified Eagle's Medium
EBSS: Earle's Balanced Salt Solution
EGF: Epidermal Growth Factor
EGFR: Epidermal Growth Factor Receptor
EMCA: Endometrial Carcinoma
EMEM: Eagle's minimum essential medium
ER: Estrogen Receptor
ERK: Extracellular signal-regulated kinases
FAT1: FAT Atypical Cadherin 1
FIGO: International Federation of Gynecology and Obstetrics
GAPDH: Glyceraldehyde-3-Phosphate Dehydrogenase
GOG: Gynecologic Oncology Group
LATS1: Large Tumor Suppressor Kinase 1
LDHA: Lactate Dehydrogenase A
LDL: Low-Density Lipoprotein
NF2: Neurofibromin 2
PBS: Phosphate Buffered Saline
PDS: Papain Dissociation System
PGK1: Phosphoglycerate Kinase 1
PPIH: Peptidylprolyl Isomerase H
PR: Progesterone Receptor
RIPA buffer: Radioimmunoprecipitation assay buffer
STAT3: Signal Transducer And Activator Of Transcription 3
TAZ: Tafazzin
TEAD: TEA Domain Transcription Factor
VP: Verteporfin
YAP: Yes-associated protein
