# Supplementary material for: Verteporfin exhibits YAP-independent anti-proliferative and cytotoxic effects in endometrial cancer cells
Source: Oncotarget. 2017 Feb 22;8(17):28628–40. doi: 10.18632/oncotarget.15614 (PMC5438678; doi:10.18632/oncotarget.15614)
Supplement: Supplementary file 1 [file oncotarget-08-28628-s001.pdf]

Verteporfin exhibits YAP-independent anti-proliferative and cytotoxic effects in endometrial cancer cells

SUPPLEMENTARY FIGURES AND TABLES

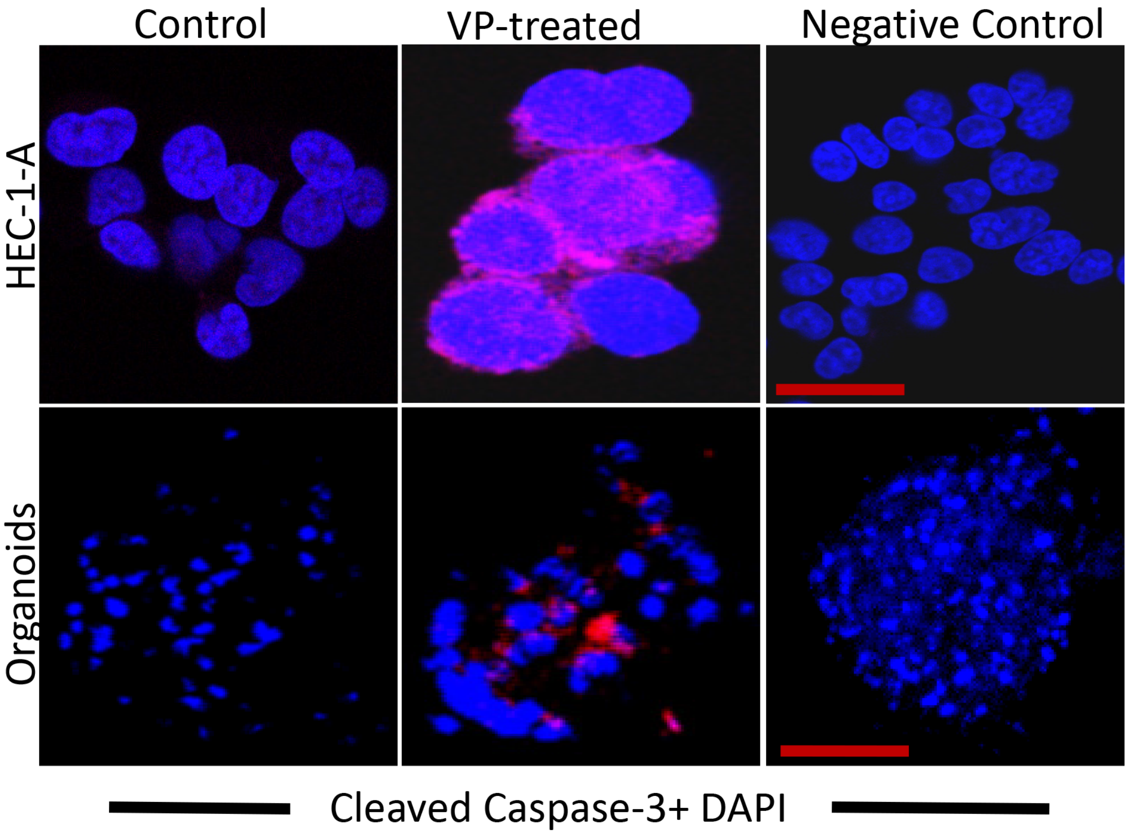

Supplementary Figure 1: VP induces caspase-3 mediated apoptosis in HEC-1-A Cells and patient derived organoids. HEC-1-A cells and patient derived organoids (#1002) were subjected to immunofluorescence detection for cleaved caspase-3 after VP treatment. Cleaved-caspase-3 (anti-rabbit) are conjugated with goat anti-rabbit Alexa flour secondary antibodies. Bar for HEC-1-A = 63x and Bar for organoids is =20x. n=3.

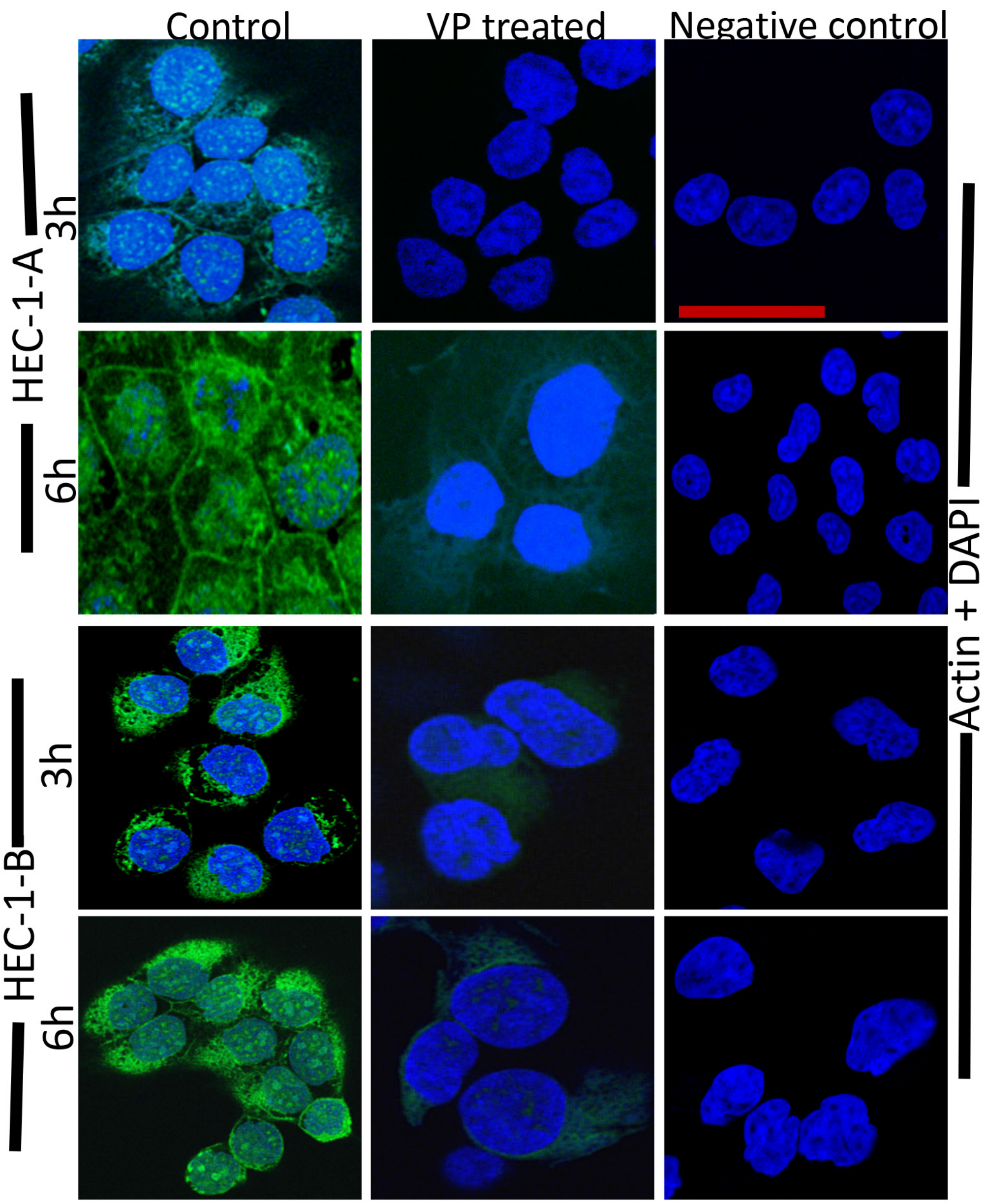

Supplementary Figure 2: VP induces phenotypic changes in EMCA cells. HEC-1-A and HEC-1-B cells were subjected to VP treatment for 3h and 6h and probed with actin antibody. This Actin antibody detects all isoforms of actin in the cells. Actin antibody (anti-goat) is conjugated with donkey anti-goat Alexa flour secondary antibodies. Bar=63x. n=3.

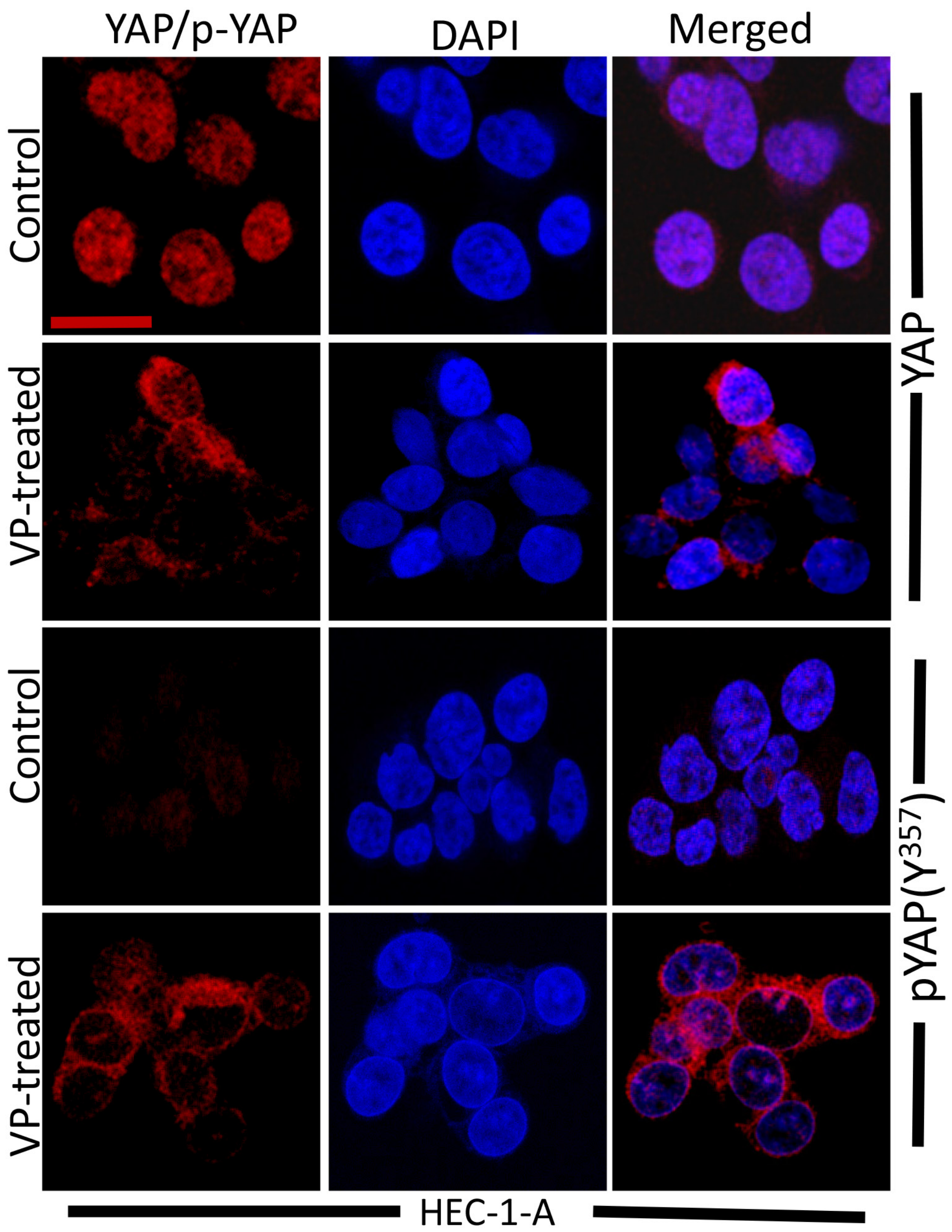

Supplementary Figure 3: VP downregulates YAP and phospho-YAP of HEC-1-A Cells. Confocal images of HEC-1-A cells that were subjected to immunofluorescence detection for YAP and phospho-YAP (Y^{357}) after VP treatment. YAP (anti-mouse) and phospho-YAP (anti-rabbit) are conjugated with goat anti-mouse and goat anti-rabbit Alexa flour secondary antibodies. Bar=63x. n=3.

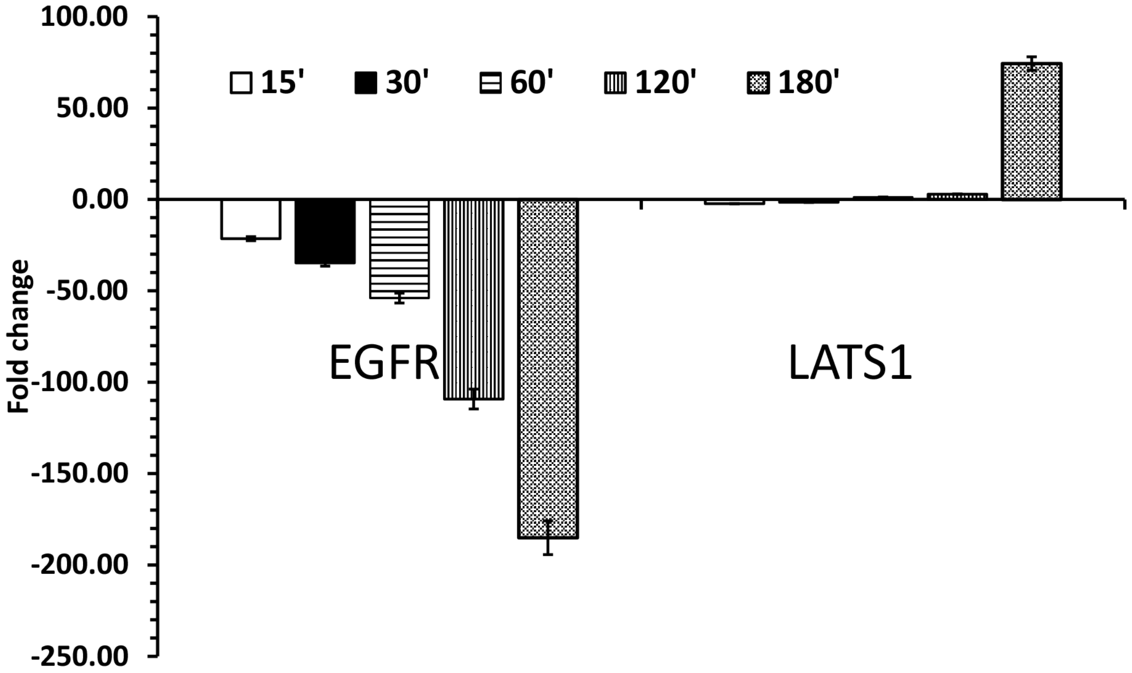

Supplementary Figure 4: RTPCR analysis of EGFR AND LATS1. Both the genes were normalized to the expression of GAPDH, β-actin, PGK1, LDHA and PPIH. Error bars indicate Mean ±SEM. For each gene, duplicates were performed from 3 different samples for each treatment. n=6.

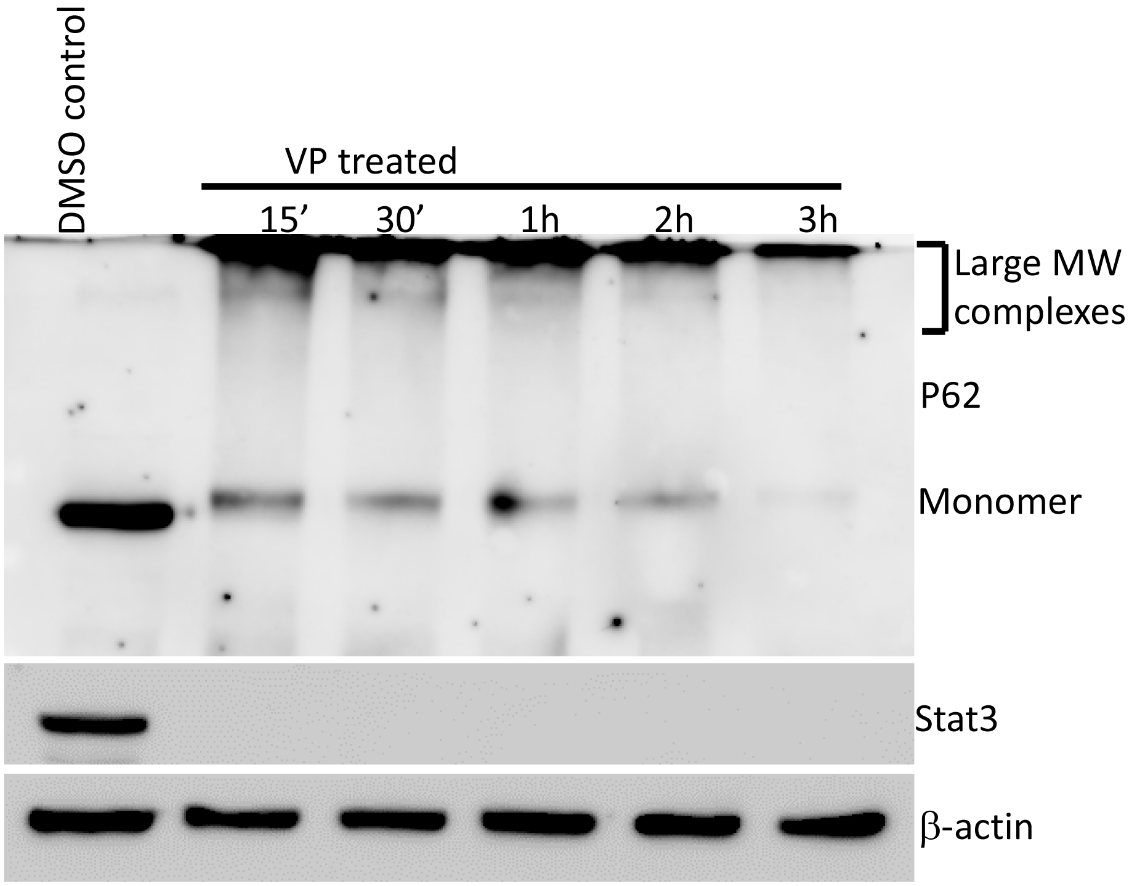

Supplementary Figure 5: Western blot time course of VP effect on p62 and Stat3. Equal amounts of proteins (40μg) from untreated and treated (10 nM VP, different time points) EMCA cells were loaded on 8% gels and transferred onto nylon membranes, which were then probed with respective antibodies. They were reprobed with β-actin which was used a positive loading control. n=3.

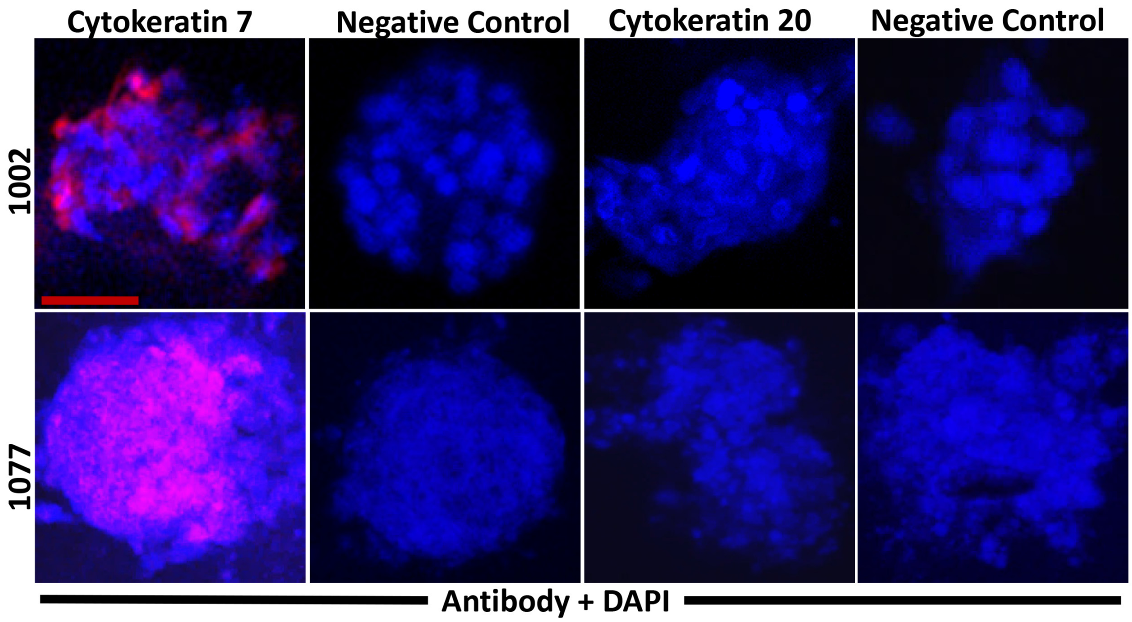

Supplementary Figure 6: Characterization of organoids based on molecular markers. Confocal images of patient derived organoids (#1002 and #1077) which were subjected to immunofluorescence detection of cytokeratin 7 (CK7) and cytokeratin 20 (CK20) in organoids. CK7 and CK20 are conjugated with goat anti-mouse and goat anti-rabbit Alexa flour secondary antibodies respectively. Bar=63x. Negative controls do not have primary antibodies. n=3.

Supplementary Table 1: Fold change of Hippo Signaling pathway genes in endometrial cancer cells after Verteporfin treatment

NM No.	Gene Description	Fold change after VP treatment			
		HEC1A		HEC1B	
		Fold change	p-value	Fold change	p-value
NM_133265	AMOT	-1.36	0.3196	2.44	0.6605
NM_057749	CCNE2	-1.35	0.3641	1.36	0.9858
NM_005245	FAT1	-3.73	0.2580	-1.86	0.4912
NM_004466	GPC5	29.47	0.5937	1.39	0.4226
NM_014240	LIMD1	1.31	0.2952	1.51	0.9492
NM_004140	LLGL1	-1.38	0.2549	2.66	0.6359
NM_002398	MEIS1	5.02	0.6852	3.29	0.5429
NM_173468	MOB1B	2.55	0.5503	1.54	0.9400
NM_020998	MST1	7.15	0.8382	-1.29	0.6566
NM_000268	NF2	-1.54	0.2548	2.30	0.6973
NM_001099771	POTEF	2.10	0.5223	1.34	0.9757
NM_014737	RASSF2	3.19	0.5397	1.62	0.9228
NM_032023	RASSF4	6.13	0.6922	3.36	0.5517
NM_182706	SCRIB	2.67	0.4117	1.44	0.9825
NM_005900	SMAD1	-2.07	0.3358	2.75	0.6109
NM_000116	TAZ	1.28	0.3162	1.80	0.8420
NM_003214	TEAD3	-1.54	0.2892	2.67	0.5952
NM_004817	TJP2	-1.73	0.2232	1.59	0.9239
NM_003722	TP63	23.60	0.9766	2.70	0.1247
NM_173485	TSHZ2	59.69	0.0466	2.2	0.4226
NM_020856	TSHZ3	10.73	0.4226	1.48	0.3815
NM_006106	YAP1	1.23	0.3554	1.78	0.8473

Results are based on cDNA PCR profiler RTPCR array experiments. n=2.
p values are based 1-way ANOVA (DMSO control vs. VP treatments).

Supplementary Table 2: Table showing details of primary antibodies used

Antigen	Type	Dilution	Manufacturer
β-actin	Mouse monoclonal	1:1000	Santa Cruz
Akt	Mouse monoclonal	1:500	Santa Cruz
p-Akt1/2/3 Antibody (Ser 473)	Rabbit monoclonal	1:300	Santa Cruz
Caspase-3	Rabbit polyclonal	1:500	Santa Cruz
Cleaved caspase-3	Rabbit polyclonal	1:500	Santa Cruz
CTGF	Mouse monoclonal	1:500	Santa Cruz
Cytokeratin-7	Mouse monoclonal	1:50	Santa Cruz
Cytokeratin-8	Rabbit polyclonal	1:50	Santa Cruz
EGFR	Rabbit polyclonal	1:300	Santa Cruz
EGFR	Mouse monoclonal	1:300	Santa Cruz
GAPDH	Mouse monoclonal	1:1000	Santa Cruz
LATS1	Rabbit polyclonal	1:500	Santa Cruz
NF2	Rabbit polyclonal	1:250	Santa Cruz
PI3K	Rabbit polyclonal	1:300	Cell Signaling Technology
RASSF1	Mouse monoclonal	1:500	LifeSpan BioSciences, Inc.
TAZ	Rabbit polyclonal	1:500	Santa Cruz
TEAD3	Rabbit polyclonal	1:250	Abcam
YAP	Mouse monoclonal	1:500	Santa Cruz
Phospho-YAP (y357)	Rabbit polyclonal	1:250	Abcam

Supplementary Table 3: Table showing details of secondary antibodies used

Type	Dilution	Manufacturer
Goat Anti-Rabbit IgG-HRP	1:5000	Boston Bioproducts
Goat Anti-Mouse IgG-HRP	1:5000	Boston Bioproducts
Alexa Flour 594 Goat Anti-Mouse IgG	1:100	Invitrogen Molecular Probes
Alexa Flour 594 Goat Anti-Rabbit IgG	1:100	Invitrogen Molecular Probes

Supplementary Table 4: Details of the primer sequences used in the study

NM No.	Gene	Forward sequence (5'→3')	Reverse sequence (5'→3')
NM_001901.2	CTGF	GCCAGAGAGTGAGAGACATTAAC	GTGAGGCTACCACATTTCCTAC
NM_000268	NF2	CTCCAGACCTAGAGCGTAAGTA	GAAGTAGACACGGCAGCTAAA
NM_032023	RASSF4	CTGCAGACAAGAGGAAGAGAAG	GTAGAAGTGGCCGTTGATAGAG
NM_000116	TAZ	CTTGCTGCCTTCTGGATTCT	TTGCTCACCTGCCTTCTATG
NM_003214	TEAD3	TTCAGACTGGGCATGAAGAAG	CTGCTACTCTAGGCAGGTAGAT
NM_001130145	YAP1	TTCCTTAACAGTGGCACCTATC	TCTGCCTGAGGGCTCTATAA
NM_004690	LATS1	CAAGGACAGAGAGGCATTAGTT	GGTATCCAAGAAGGGTGTGTAG
NM_005228	EGFR	GCTGGATGATAGACGCAGATAG	TGGGAACGGACTGGTTTATG
NM_001101	β-ACTIN	GAAGTCCCTTGCCATCCTAAA	GTCTCAAGTCAGTGTACAGGTAAG
NM_002046	GAPDH	TGATGACATCAAGAAGGTGGTGAAG	TCCTTGGAGGCCATGTGGGCCAT
NM_006347	PPIH	CACCTTCCACAGGGTCATAAA	ACTCAAGAACACCACCAAGAA
NM_000291	PGK1	GATTACCTTGCCTGTTGACTTTG	AGTGTCTCCACCACCTATGA
NM_005566	LDHA	GCCTGTGCCATCAGTATCTT	TGCAGTTCGGGCTGTATTT

All the primer sequences are based on human gene sequences. The primers were designed using PrimerQuest tool and obtained from Integrated DNA Technologies, Inc. (IDT).
